# Comprehensive analysis of *LDHAP5* pseudogene expression and potential pathogenesis in ovarian serous cystadenocarcinoma

**DOI:** 10.1186/s12935-020-01324-6

**Published:** 2020-06-10

**Authors:** Shitong Lin, Yifan Meng, Canhui Cao, Ping Wu, Peipei Gao, Wenhua Zhi, Ting Peng, Peng Wu

**Affiliations:** 1 Cancer Biology Research Center (Key Laboratory of the Ministry of Education), Tongji Hospital, Tongji Medical College, Huazhong University of Science and Technology, Wuhan, Hubei China; 2 Department of Gynecologic Oncology, Tongji Hospital, Tongji Medical College, Huazhong University of Science and Technology, Wuhan, Hubei China

**Keywords:** Ovarian serous cystadenocarcinoma, Pseudogene, *LDHAP5*, *EGFR*

## Abstract

**Background:**

We aimed to identify differentially expressed pseudogenes and explore their potential functions in four types of common gynecological malignancies (e.g., cervical squamous cell carcinoma, ovarian serous cystadenocarcinoma, uterine corpus endometrial carcinoma, and uterine carcinosarcoma) using bioinformatics technology.

**Materials and methods:**

We identified up-regulated and down-regulated pseudogenes and built a pseudogene-miRNA-mRNA regulatory network through public datasets to explore their potential functions in carcinogenesis and cancer prognosis.

**Results:**

Among the 63 up-regulated pseudogenes identified, *LDHAP5* demonstrated the greatest potential as a candidate pseudogene due to its significant association with poor overall survival in ovarian serous cystadenocarcinoma. KEGG pathway analysis revealed that *LDHAP5* showed significant enrichment in MicroRNAs in cancer, Pathway in cancer and PI3K-AKT signaling pathway. Further analysis revealed that *EGFR* was the potential target mRNA of *LDHAP5,* which may play an important role in ovarian serous cystadenocarcinoma.

**Conclusions:**

*LDHAP5* was associated with the occurrence and prognosis of ovarian serous cystadenocarcinoma, and thus shows potential as a novel therapeutic target against such cancer.

## Background

Gynecological malignancies account for a large proportion of tumors in women and seriously endanger female health. It is estimated that there will be approximately 13,800 new cases of uterine cervical cancer, 65,620 cases of uterine corpus cancer, and 21,750 cases of ovarian cancer in the United States in 2020, and with 4290, 12,590 and 13,940 possible deaths, respectively [1]. Advanced gynecological malignancies usually exhibit poor prognosis due to a lack of effective treatment in controlling distant metastasis [2]. However, most current clinical drugs are non-specific, and their therapeutic effects are limited [3]. Therefore, the identification of novel biomarkers of gynecological tumors to improve drug efficacy and prolong survival remains urgent.

The term pseudogene was first conceived by Jacp et al. [4]. Pseudogenes usually originate from paralogous functional genes (“parent gene”), but have lost the capacity to encode functional proteins due to the accumulation of mutations (e.g., frameshift mutations, early or delayed stop codons) [5]. Pseudogenes initially received little attention until *PTEN* pseudogene 1 (*PTENP1*) was found to share the same microRNA response elements (MREs) as its homologous functional parent gene, *PTEN* [6].

With the advancement of next-generation sequencing (NGS), approximately 20,000 pseudogenes have been discovered in the human genome, and the role of pseudogenes as long non-coding RNAs (lncRNAs) in the development of disease has been revealed [7–9]. Current research suggests that pseudogenes mainly regulate gene expression at the post-transcriptional level through two pathways [10]. Firstly, pseudogenes can be used as competitive endogenous RNAs (ceRNAs) to competitively bind miRNAs with the coding gene, thereby positively regulating gene expression [11–13]. For example, *PTENP1* can competitively bind miRNA-17, miRNA-21, miRNA-19, and other miRNAs through the ceRNA mechanism, thereby increasing parent gene (*PTEN*) expression by preventing miRNA-induced degradation [6]. Secondly, pseudogenes can play a negative role in the regulatory pathway, whereby they complete with their parent genes to destabilize RNA binding proteins (RBPs), resulting in a decrease in parent gene expression [14].

In the current study, we identified differentially expressed pseudogenes in four gynecological malignancies using the pseudogene database dreamBase, and then constructed a pseudogene-miRNA-mRNA regulatory network to further explore their potential functions and mechanisms in gynecological malignancies.

## Materials and methods

### Screening for dysregulated pseudogenes in four gynecological malignancies

We obtained RNA-seq data of pseudogenes in 32 human cancer from the online database dreamBase (http://rna.sysu.edu.cn/dreamBase/pancancer.php?SClade=mammal&SOrganism=hg38) [15] |Log2FC| > 2.0 was set as cutoff to identify differentially expressed pseudogenes. R v 3.5.1 and EXCEL v2016. were used to further analyze their expression landscape.

### Prognostic analysis of up-regulated expressed pseudogenes

Gene Expression Profiling Interactive Analysis (GEPIA) (http://gepia.cancer-pku.cn/) was used to evaluate prognostic values (overall survival) of up-regulated pseudogenes in 32 kinds of common human cancer [16]. The group thresholds were as follows: the group cut-off was ‘Median’, the ‘cutoff-high’ and ‘cutoff-low’ were 50%, axis units were ‘Months’, and P value < 0.05 was considered statistically significant.

### Screening for pseudogene- regulated miRNAs and miRNA-target mRNAs

The public online datasets of starBase v-2.0 and miRTarBase were used to identify pseudogene-binding miRNAs and miRNA-target mRNAs, respectively [17, 18]. The network of pseudogenes-miRNA-mRNA was constructed using Cytoscape v-3.7.2 [19].

### KEGG pathways and gene oncology (GO) enrichment analysis of target mRNAs

The list of miRNA-target genes was imported into the STRING v-11.0, and the top five significantly GO terms and KEGG pathways were selected according to the values of false discovery rate (FDR), and then were visualized by GraphPad PRISM Version 6.02 [20].

### Construction of protein–protein interaction network and identification of hub genes

STIRNG v-11.0 was used to construct the regulatory network of protein–protein, and then visualized by Centiscape plugin of Cytoscape v-3.7.2 [19–21]. The top 10 hub genes were identified according to the values of Degree unDir.

### Hub genes expression and mutations analysis

Hub genes expression and mutations analysis in ovarian serous cystadenocarcinoma were analyzed using the online cBioPortal database [22]. 489 patients (TCGA, Nature 2011) with ovarian serous cystadenocarcinoma were selected for further analysis. The select genomic profiles were as follows: ‘Mutations’; ‘Putative copy-number alterations (GISTIC)’; ‘mRNA/miRNA expression Z-scores (all genes)’, and the Z-scores threshold were ± 2. Finally, OncoPrint was obtained under the guidance of online database at c-BioPortal.

### Identification of potential target gene of *LDHAP5*

Pearson correlation analysis between *LDHAP5* and the top 10 hub genes expression in ovarian serous cystadenocarcinoma was performed using GEPIA [16]. Kaplan–Meier overall survivals of target genes were analyzed by Kaplan–Meier Plotter [23]. The mRNA expression levels of 10 hub genes in TCGA patients were further measured using Oncomine Main database [24].

## Results

### Identification of dysregulated pseudogenes in four common gynecological malignancies

**Fig. 1:**
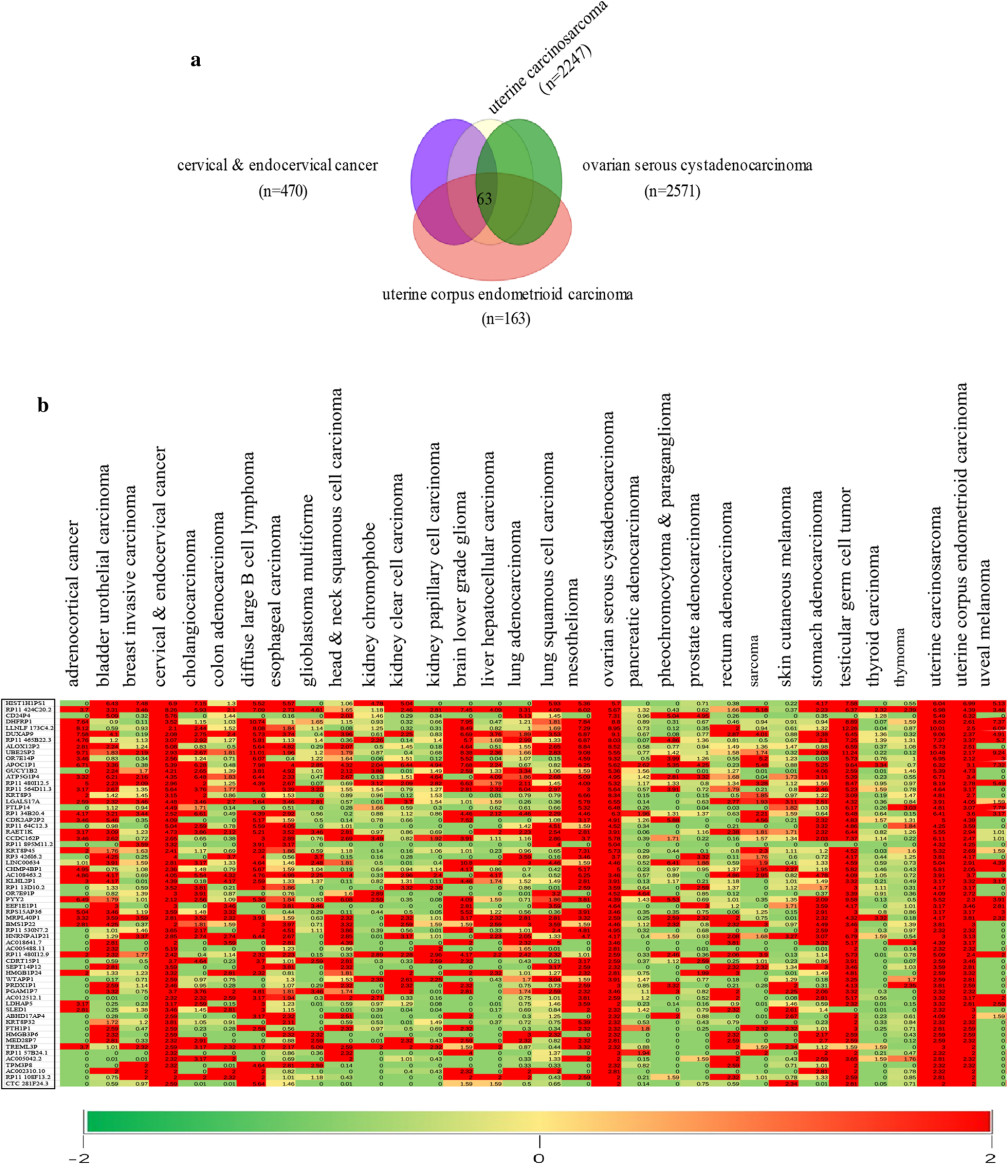
Identification of differentially expressed pseudogenes in four types of gynecological malignancies. **a** Venn diagram of 63 up-regulated pseudogenes in four gynecological malignancies. **b** Heat map of 63 frequently up-regulated pseudogenes in 32 types of human cancer. Red represents up-regulated genes and green represents down-regulated genes. Values in boxes represent |log2 FC| values

According to epidemiological statistics, cervical squamous cell carcinoma, ovarian serous cystadenocarcinoma, uterine corpus endometrial carcinoma, and uterine carcinosarcoma remain lethal diseases in women [1]. To explore the potential role of pseudogenes in carcinogenesis and cancer prognosis of four gynecological malignancies, we used the public dreamBase database to identify differentially expressed pseudogenes. As shown in Fig. 1a and Table 1, we identified 63 up-regulated and 0 down-regulated pseudogenes simultaneously in the four gynecological malignancies after preliminary screening. We then measured the expression levels of the 63 up-regulated pseudogenes in 32 types of human cancer (Fig. 1b). After removal of pseudogenes that were not highly expressed in the 32 types of human cancer, 40 pseudogenes were identified as playing potential roles in gynecological malignancies.

**Table 1:** Numbers of down-regulated pseudogenes among four types of common gynecological malignancies from dreamBase

Tumor types	Numbers of down-regulated pseudogenes
Cervical and endocervical cancer	140
Uterine carcinosarcoma	0
Ovarian serous cystadenocarcinoma	0
Uterine corpus endometrioid carcinoma	103

### Prognostic analysis of up-regulated pseudogenes in 32 types of human cancer

**Fig. 2:**
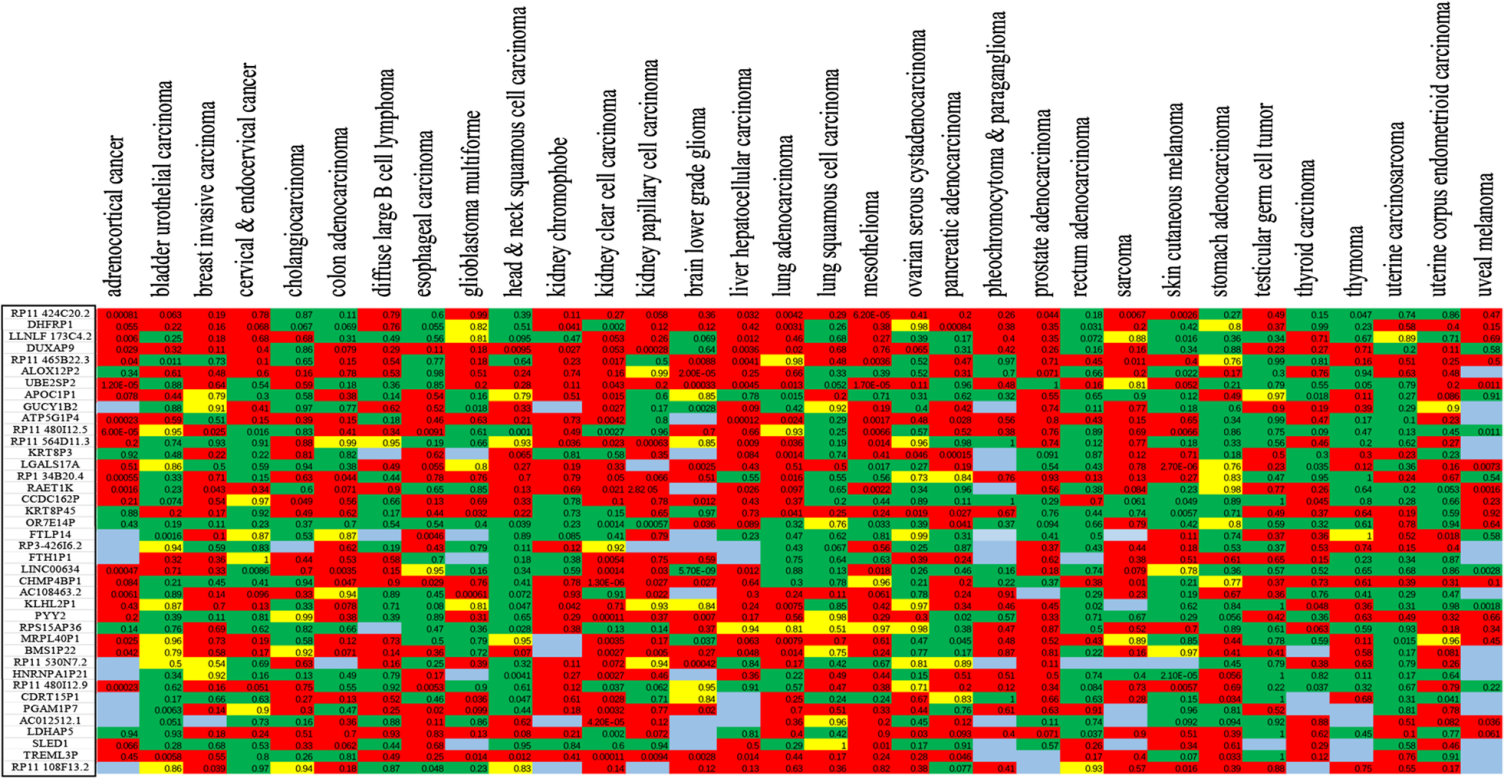
Prognostic values of 40 upregulated pseudogenes in 32 kinds of human cancer using GEPIA. Red represents poor outcome, green represents good prognosis, yellow represents neutral outcome (hazard ratio = 1), and light blue represents insufficient sample size at these custom thresholds. Values in boxes are *P*-values. *P*-values less than 0.05 were considered statistically significant. GEPIA: gene expression profiling interactive analysis

We next explored the prognostic values of the 40 up-regulated pseudogenes in the 32 kinds of human cancer using GEPIA. As shown in Fig. 2, *KRT8P3*, *KRT8P45*, and *LDHAP5* predicted poor overall survival in ovarian serous cystadenocarcinoma (HR = 1.3, *P *= 0.046; HR = 1.3, *P* = 0.019; HR = 1.3, *P *= 0.03, respectively), *FTLP14* predicted poor unfavorable prognosis in uterine corpus endometrioid carcinoma (HR = 2.6, *P* = 0.018) No other pseudogenes that were significantly correlated with poor prognosis in the four types of gynecological malignancies.

### Investigation of pseudogene-miRNA-mRNA regulatory network

**Fig. 3:**
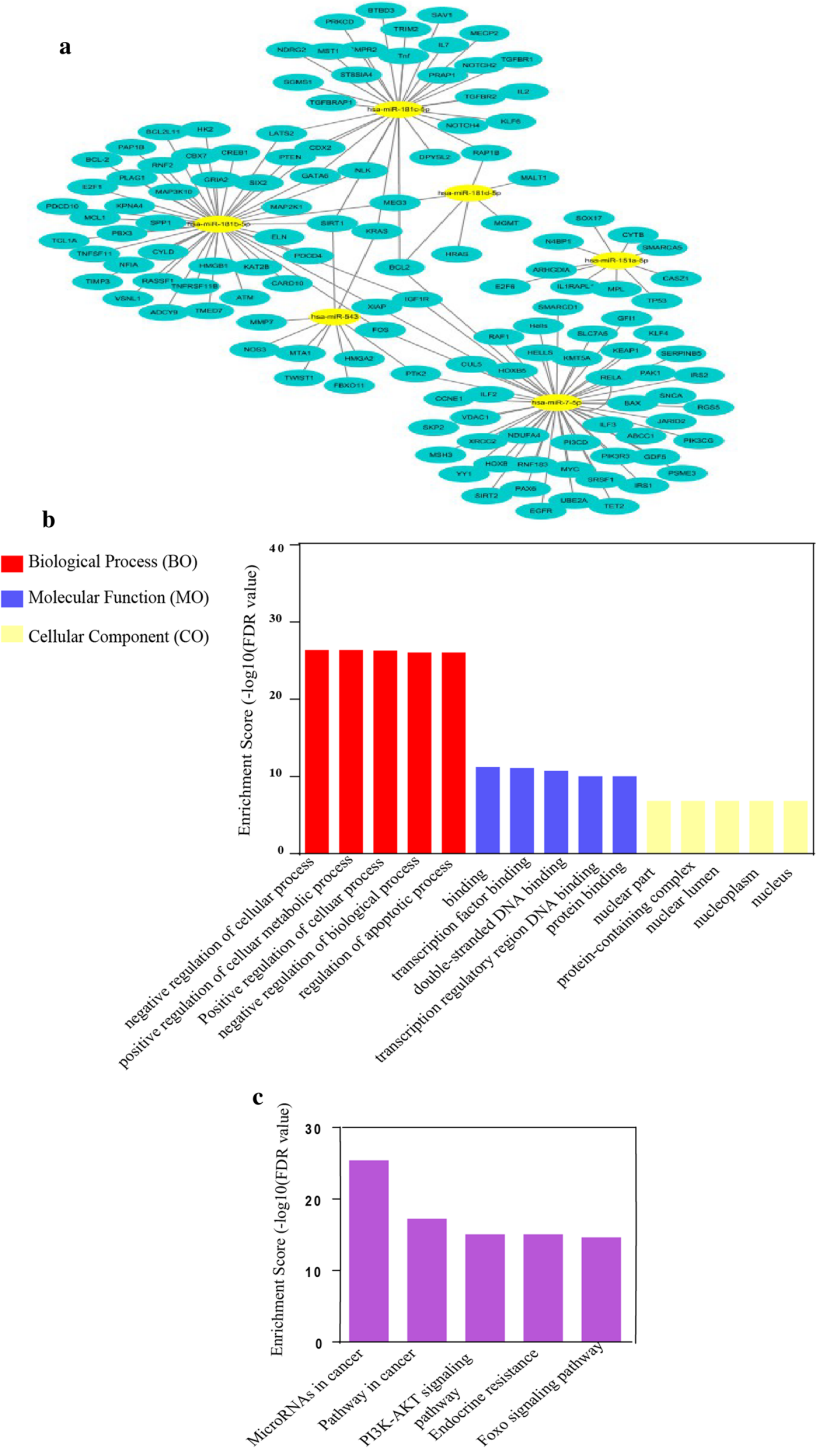
Regulatory pseudogene-miRNA-mRNA network and enrichment analysis of 148 miRNA target mRNAs. **a** Pseudogene-miRNA-mRNA network constructed by Cytoscape v-3.7.2. **b** 148 miRNA target mRNAs were divided into three functional groups: i.e., biological processes, cellular components, and molecular functions. Top five GO enriched terms are shown according to FDR values. **c** Top five KEGG pathways are shown according to the FDR values. *GO* Gene Oncology, *FDR* false discovery rate

By searching the starBase v2.0 database, only *LDHAP5* had its corresponding miRNAs. The specific characteristics of the nine retrieved miRNAs are shown in Table-S1. In addition, as shown in Table-S2, only hsa-miR-181d-5p, hsa-miR-181c-5p, hsa-miR-7-5p, hsa-miR-543, hsa-miR-151a-5p, and hsa-miR-181b-5p had their own target genes. In total, 148 miRNA target genes, which were validated by at least one of three robust method (i.e., reporter assay, western blot, and quantitative-real-time polymerase chain reaction (qRT-PCR)), were identified via miRTarBase. The pseudogene-miRNA-mRNA network was constructed using Cytoscape v_3.7.2 (Fig. 3a).

### KEGG pathway and gene oncology (GO) enrichment analysis of miRNA target mRNAs

The 148 miRNA target genes were imported into STRING v-11.0, with GO and KEGG pathway enrichment analysis then performed under the operational guidance of the website. We selected the top five significantly enriched GO terms and KEGG pathways according to false discovery rate (FDR) values. The top five Biological Process (BO), Molecular Function (MO) and Cellular Component (CO) and their corresponding FDR values are shown in Fig. 3b. The top five significantly enriched KEGG pathways were MicroRNAs in cancer (hsa05206, FDR = 4.32E−26), Pathway in cancer (hsa05200, FDR = 6.77E−18), PI3K-AKT signaling pathway (hsa04151, FDR = 9.95E−16), Endocrine resistance (hsa01522, FDR = 9.95E−16), and Foxo signaling pathway (hsa04068, FDR = 2.65E−15) (Fig. 3c). These findings confirmed that the *LDHAP5* pseudogene may mediate the occurrence and progression of ovarian serous cystadenocarcinoma.

### *EGFR* as target mRNA of *LDHAP5* in ovarian serous cystadenocarcinoma

**Fig. 4:**
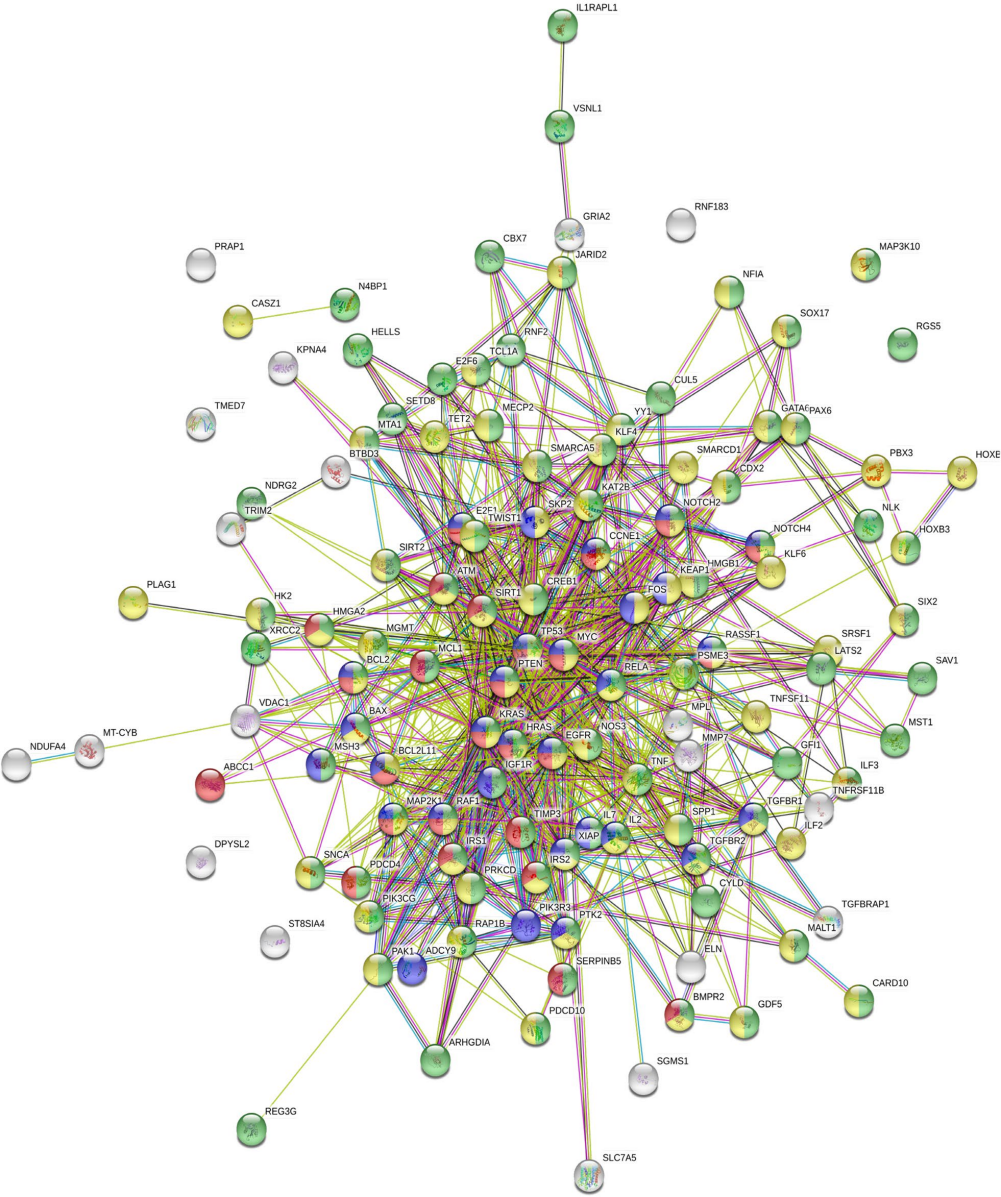
Construction of protein–protein interaction network of 148 target genes of *LDHAP5* using STRING v-11.0

**Fig. 5:**
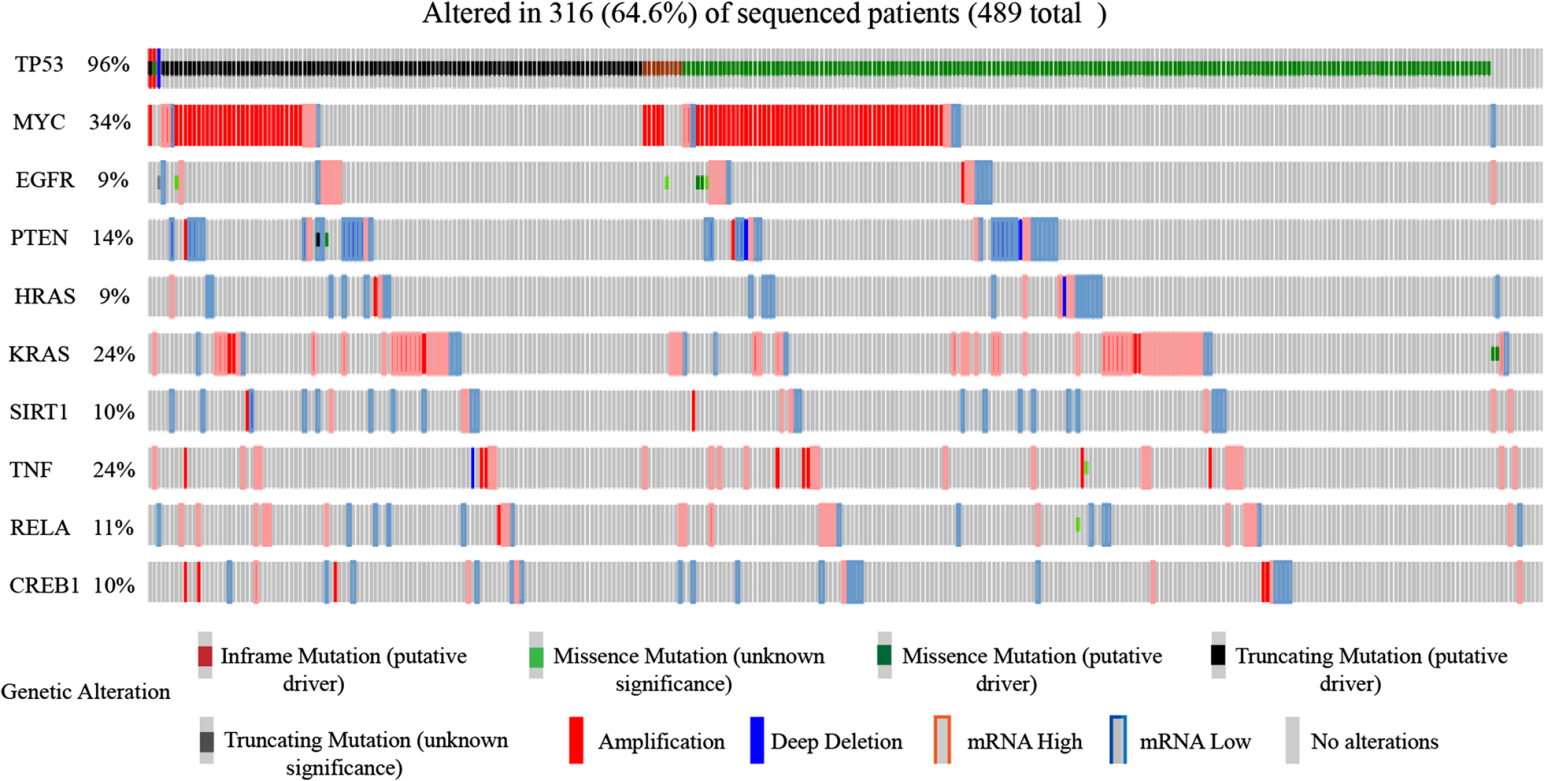
Genetic mutation analysis of 10 hub genes in ovarian serous cystadenocarcinoma (TCGA, Nature 2011). Onco-Print of c-Bioportal displays mutation types and their corresponding proportions of 10 hub genes in ovarian serous cystadenocarcinoma. TCGA: The Cancer Genome Atlas

**Fig. 6:**
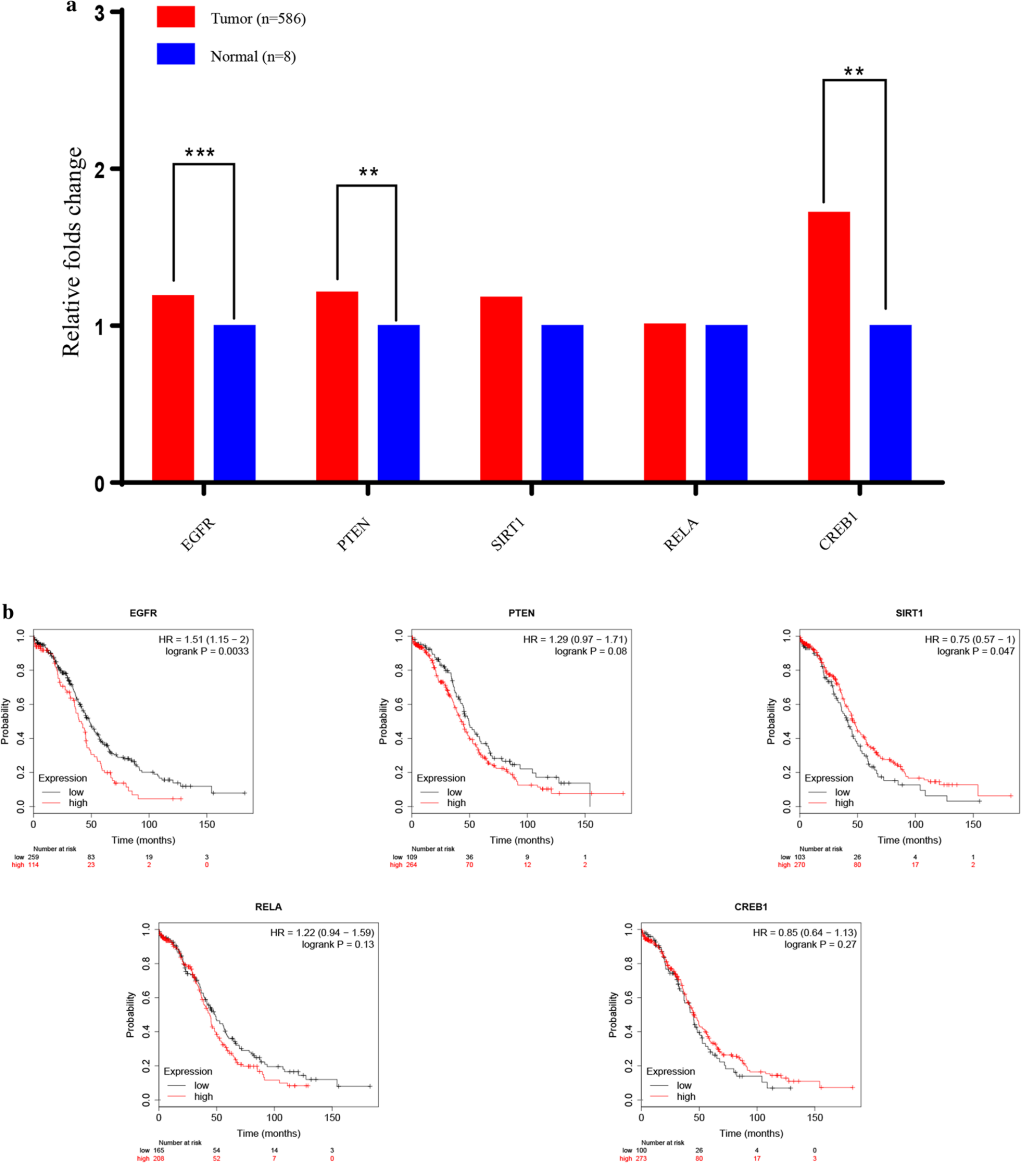
*EGFR* as the potential target gene of *LDHAP5*. **a** Expression levels of five candidate genes in TCGA ovarian samples (n = 594) using Oncomine Main database. **b** Prognostic values (overall survival) of five potential target genes in ovarian serous cystadenocarcinoma using Kaplan–Meier Plotter

We used the Centiscape plugin of Cytoscape v-3.7.2 to visualize the regulatory protein–protein network constructed using STRING v-11.0 (Fig. 4). The top 10 hub genes (i.e., *TP53*, *MYC*, *EGFR*, *PTEN*, *HRAS*, *SIRT1*, *TNF*, *RELA, KRAS,* and *CREB1*) were then identified based on Degree unDir values (Table 2). We further explored the sequence mutations and copy-number alterations of the 10 hub genes in ovarian serous cystadenocarcinoma using cBioportal. The group (TCGA, Nature 2011) which contained 489 patients was selected. However, only 361 patients (64.6%) were suitable for further analysis. The mutation frequencies of the 10 hub genes were *TP53* (96%), *MYC* (34%), *EGFR* (9%), *PTEN* (14%), *HRAS* (9%), *KRAS* (24%), *SIRT1* (10%), *TNF* (24%), *RELA* (11%) and *CREB1* (10%), respectively (Fig. 5). Pearson correlation analysis showed that *EGFR* (R = 0.16, *P* = 0.00072), *PTEN* (R = 0.098, *P* = 0.043), *SIRT1* (R = 0.094, *P* = 0.013), *RELA* (R = 0.18, *P* = 0.00013) and *CREB1* (R = 0.16, *P *= 0.00094) were significantly correlated with *LDHAP5* expression in ovarian serous cystadenocarcinoma (Table 3). Using the Oncomine Main database, only *EGFR* (fold-change = 1.192, *P* = 0.001), *PTEN* (fold-change = 1.214, *P* = 0.007), and *CREB1* (fold-change = 1.723, *P* = 1.66E−04) mRNAs were more highly expressed in TCGA ovarian patients (n = 594) than in normal patients (n = 8) (Fig. 6a). We further analyzed the prognostic values (overall survival) of the five hub genes in ovarian serous cystadenocarcinoma using Kaplan–Meier plotter (Table 4, Fig. 6b). Only *EGFR* was significantly correlated with poor outcome (HR = 1.51, 95% CI 1.15–2, *P *= 0.0033) in ovarian serous cystadenocarcinoma, whereas *SIRT1* predicted a good outcome (HR = 0.75, 95% CI 0.57–1, *P* = 0.047). Thus, according to the pseudogene-miRNA-mRNA regulatory mechanism, we concluded that *LDHAP5* may play potential roles in ovarian serous cystadenocarcinoma by targeting *EGFR*.

**Table 2:** The ten hub genes with their characters identified by cytoscape v-3.7.2

Gene name	Betweenness unDir	Closeness unDir	Degree unDir
TP53	3022.961	0.006211	77
MYC	2046.146	0.005882	67
EGFR	999.453	0.005348	53
PTEN	813.2333	0.005348	53
HRAS	604.1613	0.005291	51
KRAS	636.6394	0.005208	48
SIRT1	272.0242	0.004808	37
TNF	406.4497	0.004808	36
RELA	260.4591	0.004785	35
CREB1	479.3882	0.004651	32

**Table 3:** Pearson correlation analysis between LDHAP5 and ten hub genes expression in ovarian serous cystadenocarcinoma using GEPIA

Gene names	R	P
TP53	− 0.022	0.65
MYC	0.0044	0.93
EGFR	0.16	0.00072
PTEN	0.098	0.043
HRAS	0.089	0.065
KRAS	0.0073	0.88
SIRT1	0.094	0.013
TNF	0.038	0.43
RELA	0.18	0.00013
CREB1	0.16	0.00094

*GEPIA* Gene expression profiling interactive analysis

**Table 4:** Prognostic values of five candidate hub genes in ovarian serous cystadenocarcinoma using Kaplan–Meier plotter

Gene names	HRs with 95% CIs	P	Poor/good	FDR (%)
EGFR	1.51 (1.15–2)	0.0033	Poor	100
PTEN	1.29 (0.97–1.71)	0.08	Poor	> 50
SIRT1	0.75 (0.57–1)	0.047	Good	> 50
RELA	1.22 (0.94–1.59)	0.13	Poor	100
CREB1	0.85 (0.64–1.13)	0.27	Good	100

*CI* Confidence interval, *HR* Hazard ratio

## Discussion

With deepening research, we continue to gain a better understanding of pseudogenes. Currently, there are two major pseudogene classifications. Firstly, pseudogenes can be divided into three categories based on differences in structure and origin, i.e., duplicated, unitary, and processed pseudogenes, respectively. Duplicated pseudogenes are caused by mutations of the gene coding region or regulatory region in the process of genome DNA tandem replication or chromosome unequal exchange [25]. Unitary pseudogenes cannot be transcribed or translated because of spontaneous mutations in the coding or regulatory regions of a single copy gene with coding function [26]. Both duplicated and unitary pseudogenes are collectively called unprocessed pseudogenes. Processed pseudogenes are formed by the random integration of mRNA transcripts into cDNA and lose their normal functions due to improper insertion sites or sequence mutations [27, 28]. Secondly, pseudogenes can be classified based on their functions into pseudogenes that can be transcribed, pseudogenes that cannot be transcribed, and pseudogenes that can encode short-chain peptides or truncated proteins. These pseudogenes play important roles in carcinogenesis and cancer prognosis [29–31].

Centered on the ceRNA hypothesis, our research focused on pseudogenes that can be transcribed into mRNA. We used the pseudogene-miRNA-mRNA regulatory network to identify pseudogenes that may play potential roles in common gynecological malignancies and to explore their related mechanisms.

The initial goal of our study was to discover pseudogenes that were differentially expressed in four common gynecological malignancies. However, we only found three and one significantly up-regulated pseudogenes that predicted poor prognosis in ovarian serous cystadenocarcinoma and uterine corpus endometrioid carcinoma after Kaplan–Meier survival analysis. We selected *LDHAP5* as the candidate pseudogenes as it had corresponding miRNAs. There are two reasons accounting for the lack of pseudogenes. Firstly, many pseudogenes remain unidentified. Initially, pseudogenes were considered as “junk” or “fossil” DNA, and many methods were developed to avoid their detection [32–36]. The second possibility is that the current ceRNA hypothesis is not yet perfect, and further analysis is needed to build a more comprehensive regulatory network [37].

In our study, 148 potential target mRNAs were identified. Functional enrichment analysis showed the top five significantly enriched gene sets were MicroRNAs in cancer (hsa05206), Pathway in cancer (hsa05200), PI3K-AKT signaling pathway (hsa04151), Endocrine resistance (hsa01522), and Foxo signaling pathway (hsa04068). Interestingly, epithelial ovarian cancer, bladder cancer, lung cancer, and colorectal cancer were enriched in the MicroRNAs in cancer pathway (hsa05206). The PI3K-AKT signaling pathway has been researched extensively and plays an important role in a variety of cancers. Studies have shown that activated AKT mediates various downstream reactions, including cell survival, growth, proliferation, cell migration, and angiogenesis via phosphorylation of a range of intracellular proteins [38, 39]. More significantly, studies have shown that *EGFR* is dysregulated in many solid tumors, and PI3K-AKT signaling can be used as a downstream regulatory pathway for *EGFR* to mediate the occurrence and progression of disease, as confirmed in many cancers [40, 41].

Our research has several limitations. Specially, our conclusions are primarily based on the analysis of existing databases. To further confirm the role of the *LDHAP5* pseudogene at the in vivo and in vitro level, we need to construct ovarian cancer cell lines that differentially express *LDHAP5*, with clinical pathological specimens from ovarian cancer patients also used to verify our findings. *EGFR* antagonists (e.g., gefitinib, lapatinib, erlotinib) have been used in a variety of cancers, including pancreatic, small cell lung, and colorectal cancer [42–44]. Once our research is successfully validated, it may be used in ovarian cancer in the future. With continuing research, more pseudogene functions and corresponding mechanisms will be revealed, which could help in the identification of novel biomarkers, development of specific drug design, and the adoption of personalized treatment in the future.

## Conclusions

This study is the first to report on the high expression of the *LDHAP5* pseudogene in ovarian serous cystadenocarcinoma, which may lead to poor prognosis via its targeting of *EGFR*. Thus, *LDHAP5* may serve as a new therapeutic target, and improve the prognosis of patients with ovarian cancer in the future.

## Supplementary information

**Additional file 1: Table S1.** miRNAs targeting LDHAP5 were predicted by starBase v2.0.

**Additional file 2: Table S2.** Numbers of miRNA target gene identified by miRTarBase.

## Data Availability

Not applicable.

## Abbreviations

MREs: MicroRNA response elements
lncRNAs: Long non-coding RNAs
ceRNAs: Competitive endogenous RNAs
RBPs: RNA binding proteins
GEPIA: Gene expression profiling interactive analysis
GO: Gene oncology
FDR: False discovery rate
