# Supplementary material for: Comprehensive analysis of LDHAP5 pseudogene expression and potential pathogenesis in ovarian serous cystadenocarcinoma
Source: Cancer Cell Int. 2020 Jun 10;20:229. doi: 10.1186/s12935-020-01324-6 (PMC7288418; doi:10.1186/s12935-020-01324-6)
Supplement: Supplementary file 1 — Additional file 1: Table S1. miRNAs targeting LDHAP5 were predicted by starBase v2.0. [file 12935_2020_1324_MOESM1_ESM.docx]

**Table S1. miRNAs targeting LDHAP5 were predicted by starBase v2.0.**

| miRNA names | mirAccession | Pseudogene name | Targetsites | BioComplex | ClipReeadnum |
| --- | --- | --- | --- | --- | --- |
| hsa-miR-181b-5p | MIMAT0000257 | LDHAP5 | 1 | 2 | 2 |
| hsa-miR-151a-5p | MIMAT0000256 | LDHAP5 | 1 | 2 | 2 |
| hsa-miR-3167 | MIMAT0015042 | LDHAP5 | 1 | 2 | 2 |
| hsa-miR-543 | MIMAT0004954 | LDHAP5 | 1 | 2 | 2 |
| hsa-miR-7-5p | MIMAT0000252 | LDHAP5 | 1 | 2 | 2 |
| hsa-miR-181c-5p | MIMAT0000258 | LDHAP5 | 1 | 2 | 2 |
| hsa-miR-181d-5p | MIMAT00002821 | LDHAP5 | 1 | 2 | 2 |
| hsa-miR-4262 | MIMAT0016894 | LDHAP5 | 1 | 2 | 2 |
| hsa-miR-876-5p | MIMAT0004924 | LDHAP5 | 1 | 2 | 2 |
