# Supplementary material for: Comprehensive analysis of LDHAP5 pseudogene expression and potential pathogenesis in ovarian serous cystadenocarcinoma
Source: Cancer Cell Int. 2020 Jun 10;20:229. doi: 10.1186/s12935-020-01324-6 (PMC7288418; doi:10.1186/s12935-020-01324-6)
Supplement: Supplementary file 2 — Additional file 2: Table S2. Numbers of miRNA target gene identified by miRTarBase. [file 12935_2020_1324_MOESM2_ESM.docx]

**Table S2 . Numbers of miRNA target gene identified by miRTarBase.**

| miRNA names | mirAccession | Pseudogene name | Numbers of target genes |
| --- | --- | --- | --- |
| hsa-miR-181b-5p | MIMAT0000257 | LDHAP5 | 44 |
| hsa-miR-151a-5p | MIMAT0000256 | LDHAP5 | 10 |
| hsa-miR-3167 | MIMAT0015042 | LDHAP5 | 0 |
| hsa-miR-543 | MIMAT0004954 | LDHAP5 | 9 |
| hsa-miR-7-5p | MIMAT0000252 | LDHAP5 | 50 |
| hsa-miR-181c-5p | MIMAT0000258 | LDHAP5 | 30 |
| hsa-miR-181d-5p | MIMAT00002821 | LDHAP5 | 6 |
| hsa-miR-4262 | MIMAT0016894 | LDHAP5 | 0 |
| hsa-miR-876-5p | MIMAT0004924 | LDHAP5 | 0 |
